# 2205. Utilization of guiding comments imbedded within culture results to improve antimicrobial prescribing for enterococcal urinary tract infections in the ambulatory setting

**DOI:** 10.1093/ofid/ofad500.1827

**Published:** 2023-11-27

**Authors:** Julia Pepper, Aundrea Rosenberger, Raghavendra Tirupathi, Jarett Logsdon

**Affiliations:** Wellspan Waynesboro Hospital, Waynesboro, Pennsylvania; Wellspan York Hospital, York, Pennsylvania; Keystone Health, Chambersberg, Pennsylvania; WellSpan Health, Waynesboro, Pennsylvania

## Abstract

**Background:**

Evaluate outcomes associated with utilizing guiding comments imbedded within the electronic health record’s (EHR) culture result to decrease the inappropriate use of cephalosporins in enterococcal urinary tract infections.

**Methods:**

Data collected retrospectively from a regional health system from 02/01/22 to 02/28/23. Inclusion criteria was urine culture positive for enterococcus and active cephalosporin order. Exclusion criteria was cephalosporin treatment prescribed for a non-enterococcal organism and age less than 18 years. Patients were evaluated 6 months pre- and post-implementation of a microbiology comment added to streptococcus gamma and enterococcal cultures: “Enterococcus sp. are resistant to cephalosporins.” The primary outcome was rate of inappropriate prescribing of cephalosporins in enterococcal urinary tract infections in the ambulatory care setting. The secondary outcome was rate of 30-day follow-up visits required pre- and post-implementation of guiding comment. Follow-up was defined as either ambulatory visit, telephone visit, emergency department admission, or inpatient admission. Chi-Squared statistical test was utilized to compare nominal data.

**Figure:**
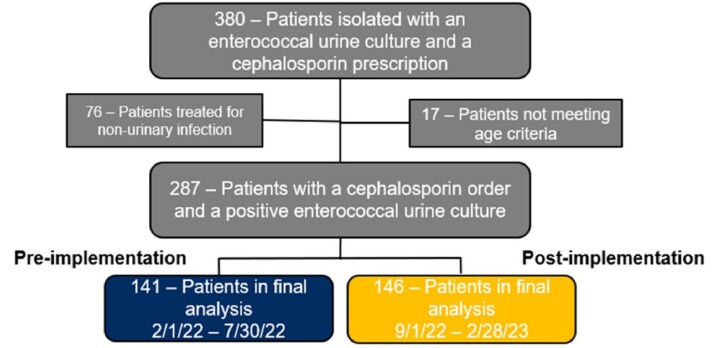

**Figure:**
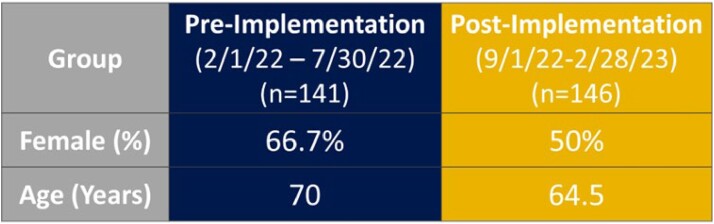

**Results:**

93 patients were not included based on inclusion and exclusion criteria. 141 patients in the pre group and 146 patients in the post group were analyzed. After guiding comment implementation, the incidence of inappropriate treatment continuation decreased from 67.4% to 43.8% (RRR=35%, p=0.001). Follow up events included patient encounters related to urinary symptoms such as emergency department admissions, inpatient admission, ambulatory visits, and telephone encounters. Total patients requiring follow-up decreased from 44.7% (63/141) to 26.0% (38/146); 41.8% relative risk reduction (p = 0.001).

**Figure:**
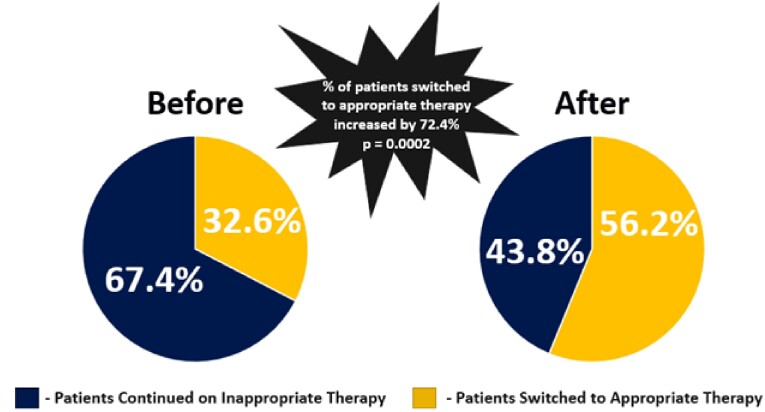

**Figure:**
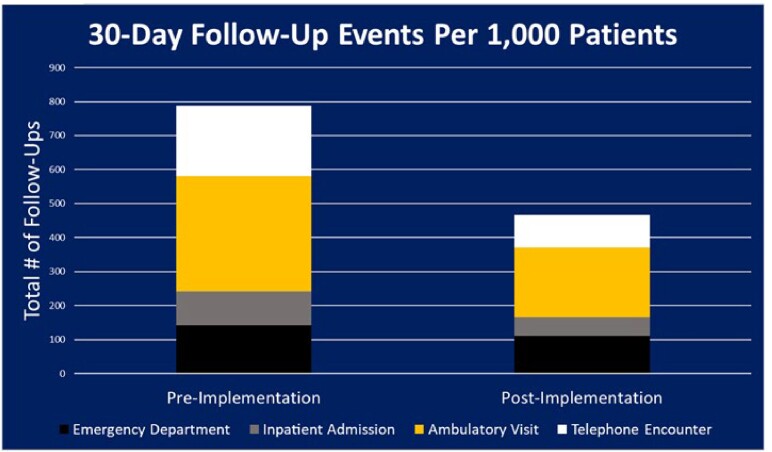

**Conclusion:**

Providers assume enterococcus sensitivity to ampicillin equates to sensitivity to all beta-lactams. The implementation of guiding comments results significantly reduced the inappropriate use of cephalosporins to treat enterococcal urinary tract infections. Total follow-up visits were also significantly reduced which has the potential to improve patient experience. Enterococcal guiding comments should be implemented at heath care facilities when possible.

**Disclosures:**

**All Authors**: No reported disclosures

